# Ancient globetrotters—connectivity and putative native ranges of two cosmopolitan biofouling amphipods

**DOI:** 10.7717/peerj.9613

**Published:** 2020-07-28

**Authors:** Jan Beermann, Allison K. Hall-Mullen, Charlotte Havermans, Joop WP Coolen, Richard PMA Crooijmans, Bert Dibbits, Christoph Held, Andrea Desiderato

**Affiliations:** 1 Department of Functional Ecology, Alfred Wegener Institute, Helmholtz Centre for Polar and Marine Research, Bremerhaven, Germany; 2 Helmholtz Institute for Functional Marine Biodiversity, Oldenburg, Germany; 3 FB2, University of Bremen, Bremen, Germany; 4 Helmholtz Young Investigator Group “Arctic Jellies”, Alfred Wegener Institute Helmholtz Centre for Polar and Marine Research, Bremerhaven, Germany; 5 Wageningen Marine Research, Den Helder, The Netherlands; 6 Chair group Aquatic Ecology and Water Quality Management, Wageningen University, Wageningen, The Netherlands; 7 Animal Breeding and Genomics, Wageningen University, Wageningen, The Netherlands; 8 Programa de Pós‐Graduação em Zoologia (PPGZOO), Departamento de Zoologia, Universidade Federal do Paraná, Curitiba, Brazil

**Keywords:** Amphipoda, Biofouling, Biological invasion, Cosmopolitan distribution, Marine dispersal, Marine shipping

## Abstract

The geographic distributions of some coastal marine species have appeared as cosmopolitan ever since they were first scientifically documented. In particular, for many benthic species that are associated with anthropogenic substrata, there is much speculation as to whether or not their broad distributions can be explained by natural mechanisms of dispersal. Here, we focused on two congeneric coastal crustaceans with cosmopolitan distributions—the tube-dwelling amphipods *Jassa marmorata* and *Jassa slatteryi*. Both species are common elements of marine biofouling on nearly all kinds of artificial hard substrata in temperate to warm seas. We hypothesized that the two species’ modern occurrences across the oceans are the result of human shipping activities that started centuries ago. Mitochondrial DNA sequences of the CO1 fragment of specimens from distinct marine regions around the world were analysed, evaluating genetic structure and migration models and making inferences on putative native ranges of the two *Jassa* species. Populations of both species exhibited considerable genetic diversity with differing levels of geographic structure. For both species, at least two dominant haplotypes were shared among several geographic populations. Rapid demographic expansion and high migration rates between geographically distant regions support a scenario of ongoing dispersal all over the world. Our findings indicate that the likely former native range of *J. marmorata* is the Northwest Atlantic, whereas the likely former native range of *J. slatteryi* is the Northern Pacific region. As corroborated by the genetic connectivity between populations, shipping still appears to be the more successful vector of the two species’ dispersal when compared to natural mechanisms. Historical invasion events that likely started centuries ago, along with current ongoing dispersal, confirm these species’ identities as true “neocosmopolitans”.

## Introduction

Human-mediated dispersal of organisms can exceed natural mechanisms, carrying species over large distances and often across natural barriers (Ashton et al., 2007; Cohen & Carlton, 1998; Galil, Froglia & Noël, 2002; Gollasch, 2002; Molnar et al., 2008). For example, for as long as vessels have navigated oceans across the globe, non-native marine species have been transported and introduced into new regions as part of the fouling communities on vessel hulls and, for the last 100± years, in ballast water (Carlton, 1985; Thiel et al., 2003).

Thanks to the rise of scientific documentation and description, human-mediated introductions of marine species across the globe within the last half-century have been increasingly documented. In clear contrast, the dispersal of marine species in earlier centuries is nearly undocumented and probably a great many introductions since the 1820’s have yet to be recognized. Many coastal benthic species that occur in anthropogenic habitats, especially those associated with the hulls of wooden ships (i.e., fouling and boring), had probably been distributed across the oceans by human shipping activities centuries before their geographic distributions were scientifically documented for the first time. As a consequence, modern geographic distributions of some marine species show cosmopolitan occurrences (e.g., Conlan, 1990; Thiel & Gutow, 2005), classified as “neocosmopolitan” (sensu Darling & Carlton, 2018). There is much speculation as to the likely former native ranges of coastal benthic species, with many still not reliably assigned native or introduced status in parts of their global range (Geller, Darling & Carlton, 2010). In parallel, there is mounting evidence from modern taxonomy and molecular research studies which increasingly cast doubt on the existence of marine benthic “eucosmopolitan” species (sensu Darling & Carlton, 2018; Havermans, 2016).

Here, we focused on two congeneric coastal species that are widely distributed in the world’s oceans: the tube-dwelling amphipod crustaceans *Jassa marmorata* Holmes, 1905 and *Jassa slatteryi* Conlan, 1990. Despite both species being common elements of marine fouling communities in harbors ranging from temperate to warm seas, their geographic distributions are not well characterized (Marchini & Cardeccia, 2017). *Jassa* species are effective colonizers of clean hard substrata through immigration of nearby adult animals, but primarily by recruitment of drifting juveniles through the water column (Beermann, 2014; Franz & Mohamed, 1989; Havermans et al., 2007). By these means, *Jassa* species can dominate fouling communities particularly on artificial hard substrates such as navigational buoys, harbour walls, offshore wind farms and ship wrecks, easily reaching densities of more than 1,000,000 individuals/m^2^ and exceeding other taxa in terms of abundance and biomass (Coolen et al., 2018; Franz, 1989; Scinto et al., 2007; Zintzen et al., 2008).

The long-standing difficulties in the taxonomy of the genus *Jassa* led to misidentifications and thus, unreliable and questionable species records (Conlan, 1989, 1990). Furthermore, with no historical documentation of the species’ native ranges prior to scientific documentation, it was next to impossible to retrace the origin and native ranges of these species. Although the historical invasion pathways of *J. marmorata* and *J. slatteryi* are difficult to reconstruct, given their ecology, we hypothesized that the current “cosmopolitan” distribution of these species is probably the result of “ancient” invasive events (i.e., prior to scientific documentation and description), mediated by transoceanic shipping activities that started centuries ago.

Molecular approaches have often been used to retrace species’ native ranges, particularly in the case of ecologically harmful invasive invertebrate species such as woodwasps (e.g., Boissin et al., 2012) and ladybird beetles (e.g., Lombaert et al., 2011). By using mitochondrial sequence data or shorter fragments such as microsatellites or SNPs, the origins and pathways of invasion or colonization can be retrieved, even in cases of complex histories, independent introductions and subsequent admixtures (e.g., Estoup & Guillemaud, 2010). To this purpose, we analysed mitochondrial DNA sequences of the CO1 fragment of the two globally distributed *Jassa* species in order to evaluate their genetic structure and infer their putative former native ranges.

## Materials and Methods

### Specimen collection and taxonomic identification

Samples were collected opportunistically from artificial substrates (e.g., harbor walls, pontoons, offshore constructions) within distinct marine regions via different sampling methods. A total of 419 specimens were collected, 291 of which were identified morphologically as *J. marmorata* and 128 of which were identified as *J. slatteryi*, following the original descriptions provided by Conlan (1990) (Table 1).

**Table 1:** Sampling by region and species.

Region	Population	Sampling location	Date	*J. marmorata*		*J. slatteryi*		Collector
				Total no.	Sequ. no.	Total no.	Sequ. no.	
Northern European seas	Germany	Südhafen, Helgoland	12.06.2017	7	25			J. Beermann
		Südmole, Helgoland	12.06.2017	8				
		Ponton #44, Helgoland	12.06.2017	3				
		Bollwerk 2, Helgoland	12.06.2017	2				
	North Sea	Godewind.28		28	131			R. Krone
		OWF BARD1		1				
		FINO3 structure		2				
		Riffgat wind turbine R20		54				
		Riffgat wind turbine R28		5				
		Horns rev trubine G7		41				
	Iceland	Grindavik	16.08.2018	20	20			J. Beermann
	Norway	Frei	04.08.2017	16	16			V. Fernández-Gonzalez
	Atlantic Spain/ Lusitanian	Vigo	01.08.2018	21	12	21	12	P. Domingues
		A Graña	07.05.2011			10		J. Guerra-García
		Gijon	03.05.2011			7		
		Puerto America, Cadez	14.02.2011			10		
South West Atlantic								
	Argentina	Mar del Plata Port	29.11.2016	10	10			C. Rumbold
North West Atlantic								
	USA	New Haven, Connecticut	12.07.2016	12	8			S. Jungblut
North West Pacific								
	Japan	Otsuchi Bay, Iwate	27.06.2016			10	10	M. Kodama
	South Korea	Impo, Yeosu-si	23.06.2011			6	7	Y. H. Kim
		Muchangpo	28.09.2007			6		
		Dejuk Island	26.06.2014	2		3		
Mediterranean Sea								
	Mediterranean Spain	Malaga	31.07.2014			10	15	V. Fernández-Gonzalez
		Agua Dulce, Almeria	05.29.2014			8		
		San Pedro del Pinatar	30.05.2017	5	19			
		Campello	30.05.2017	7				
		Algeciras Bay	30.01.2017	13				J. Guerra-García
		Puerto Palma Mallorca Balearic Islands	22.01.2012	2		8		
	North Africa	Ceuta	11.10.2015			10	5	
South East Pacific								
	Chile	Coquimbo	13.08.2018	15	15	17	17	M. Thiel
	Peru	Melchorita, Cañete	01.04.2017	11	8	3	1	A. Jiménez Campeán

### DNA isolation, amplification and sequencing

If available, at least 10 specimens (including males, females and juveniles) from each sampling location were used for molecular analysis. Two to three pereopods (pereopods 5–7, depending on the animal’s size) of each specimen were used for DNA isolation. DNA extractions were performed using the QIAmp DNA Mini Kit (Qiagen, Hilden, Germany) according to the manufacturer’s protocols. Polymerase chain reaction (PCR) amplifications at the cytochrome c oxidase 1 fragment were carried out using the universal primers LCO1490 (5′ GGTCAACAAATCATAAAGATATTGG 3′) and HCO2198 (5′ TAAACTTCAGGGTGACCAAAAAATCA 3′) (Folmer et al., 1994). The 25 µl reaction mix consisted of 0.2 mM dNTPs, 0.5 µM forward and reverse primers, 10 µM PCR buffer, 0.02U/µl Hotmaster Taq (5 Prime GmbH, Hamburg, Germany), 3 µl template DNA, and mol grade water to bring the mix to the final volume of 25 µl. PCR thermal cycling conditions for amplifying CO1 sequences consisted of an initial denaturation at 94 °C for 2 min, followed by 36 cycles of 94 °C for 20 s, primer annealing at 42 °C for 20 s, extension at 65 °C for 1 min, and a final extension at 65 °C for 15 mins. The PCR product quality was assessed on a 2% agarose gel, sequencing was performed at a contracting sequencing facility (EUROFINS, Germany). Material contributed by J. Coolen was processed using the same protocol as in Luttikhuizen et al. (2019).

### Sequence editing and alignments

The software CodonCode Aligner v.8.0 (CodonCode Corporation, Deham, MA, USA) was used to check electropherograms for ambiguous bases, remove primers, and to check for stop codons to prevent inclusion of pseudogenes in the analyses. Clustal W (Thompson, Higgins & Gibson, 1994) implemented in MEGA 7.0 (Kumar, Stecher & Tamura, 2016) was used to align the resulting sequences and the basic local alignment search tool (BLAST) provided by the National Center for biotechnology information website (http://blast.ncbi.nlm.nih.gov/Blast.cgi) was used as a sequence control. DNA barcode data from all genetically analyzed specimens were made publicly available in the project “JASSA” in the Barcode of Life Data System (BOLD; Ratnasingham & Hebert, 2007) (dx.doi.org/10.5883/DS-JASSA). In addition to the resulting 330 newly edited CO1 sequences (199+131) for the two species obtained for this study, 128 previously published sequences of *J. marmorata* and 16 sequences of *J. slatteryi* were retrieved from GenBank and BOLD. Thus, the complete dataset consisted of 458 DNA barcodes. Additionally, 44 haplotype sequences of *J. herdmani* (Walker, 1893) from a previous work (Luttikhuizen et al., 2019) were added as outgroup (GenBank accession numbers: MH052599–MH052642). Detailed information on the specimens and sequences are provided in Table S1.

The full alignment of 657 base pairs (bp) was collapsed to unique haplotypes, subsequently treating gaps as missing data using ALTER (Glez-Peña et al., 2010). A shorter alignment of 589 bp which excluded sequences shorter than 500 bp was chosen for the population analyses that tend to be sensitive to missing data. Finally, an alignment for each species was produced manually, preserving rare haplotypes and deleting shorter sequences of common haplotypes.

Intra- and interspecific species distances were calculated in MEGA 7.0 based on the Kimura-two-parameter (K2P) model with pairwise deletion using the full length of 657 bp sequences; these were then used to estimate genetic divergence between taxa. The K2P model was preferred over more complex and fitting models in order to ensure comparability of the results with those of published literature and other species delimitation analyses (Del Pasqua et al., 2018).

In the program DNASP 5.10 (Librado & Rozas, 2009), metrics of genetic diversity within populations of at least three sequences per region and species were estimated by computing indices such as number of haplotypes (H), haplotype diversity (Hd), and nucleotide diversity (π).

To test for departure from neutrality, Fu (1997), Tajima (1989) and mismatch distribution were computed in ARLEQUIN 3.5 (Excoffier & Lischer, 2010) for three different spatial scales (population, region and species). The mismatch distribution was tested against the predicted outcome of the models under demographic and spatial expansion in order to give further support to the neutrality tests. Departure from neutrality as well as demographic and spatial expansion would support the hypothesis of neocosmopolitanism whereas stability would support an eucosmopolitan scenario. Fixation indices were used to analyze genetic differentiation between populations (Fst), among regions of populations (Fct), and among populations within regions (Fsc; Wright, 1949). The Fst values were visualized in non-metric multidimensional scaling (nMDS) plots for each species in PRIMER 6 (v. 1.0.3). The significance of uncorrected pairwise Fst values were tested by performing 10,100 permutations in ARLEQUIN with the null hypothesis of no differentiation.

In order to test for a possible differentiation among regions or populations, one-way and two-way analyses of molecular variance (AMOVA; Excoffier, Smouse & Quattro, 1992) were performed in ARLEQUIN with 10,100 permutations for each species. A high degree of differentiation would suggest low connectivity and low genetic flow between populations, disproving the hypothesis of a neocosmopolitan distribution.

As possible differences between populations can be related to the remoteness of some locations, the relationship between geographic and genetic distances was tested with a Mantel test implemented in Alleles in Space (AIS; Miller, 2005) with 10,000 permutations. Geographic distances were calculated between coordinates regardless of geographic barriers (such as continents, currents). This was set to provide good approximation and strength to the other tests and to exclude simple spatial correlation.

In addition to a putative poor differentiation, a high degree of migration between populations can provide further information on the present connectivity between populations, supporting a neocosmopolitan scenario. Migration rate among regions was assessed using MIGRATE-N 3.6.11 (Beerli, 2006) on the CIPRES Science Gateway (Miller, Pfeiffer & Schwartz, 2010). By using a coalescent approach and Bayesian Markov chain Monte Carlo (MCMC) method, MIGRATE-N quantified both population size (θ = 2N_e_μ for haploid mtDNA) and migration rate (M = m/μ). The product is the number of effective migrants per generation (2N_e_m). The probabilities were estimated using 10 replicates, wherein each replicate was run for 50,000,000 generations and sampled every 1,000 steps, thereby recording a total of 50,000 steps. Each replicate included four MCMC chains with relative temperatures of 1.0, 1.5, 3.0 and 100,000. Convergence was assessed to ensure that the effective sample size (ESS) was higher than 200. Migration rates were only considered when 95% of the confidence intervals (CIs) were different from zero.

A haplotype network was computed for each species using an alignment of 372 sequences and 636 bp for *J. marmorata*, and 85 sequences and 595 bp for *J. slatteryi* with the median-joining method (Bandelt, Forster & Röhl, 1999) in the software Popart (v. 1.7).

### Phylogenetic analyses and ancestral range estimations

Phylogenetic relationships among the *Jassa* species were reconstructed using only unique haplotypes with maximum likelihood (ML) and Bayesian inference (BI) of the CO1 gene fragment. The ML was inferred with PhyML (Guindon et al., 2010) using 1,000 bootstraps for the branch support. The best substitution model (GTR + I + G) was tested with the SMS routine in PhyML using both AIC and BIC as optimality criteria (Lefort, Longueville & Gascuel, 2017).

A time-calibrated BI phylogeny was reconstructed in BEAST 2.5.2 (Bouckaert et al., 2014) on XSEDE (Towns et al., 2014). The GTR model of evolution, with proportion of invariant (I) and gamma shape parameters (G), determined with bModelTest (Bouckaert & Drummond, 2017), and the Yule speciation model were set for priors. An uncorrelated relaxed clock with log-normal distribution was applied following the specifications proposed by Copilaş-Ciocianu, Sidorov & Gontcharov (2019); that is, rates ranged from 0.7% to 1.7% Ma^−1^ with a starting value of 1.2%. Three runs each of 20,000,000 iterations of MCMC sampled each 1,000 iterations were performed. All runs were examined using Tracer v1.7.1 and all sampled parameters achieved sufficient sample sizes (ESS > 200). Tree files were combined using LogCombiner on XSEDE (1.8.4) with 15% of burn-in. The maximum clade credibility tree was generated using TreeAnnotator on XSEDE. All the XSEDE analyses were performed on the CIPRES Science Gateway (Miller, Pfeiffer & Schwartz, 2010).

The ancestral ranges of the haplotypes were estimated with the R package BioGeoBears (Matzke, 2013; R Development Core Team, 2018). This package is used to perform biogeographic inferences such as the estimation of ancestral ranges of species or Operational Taxonomic Units (OTUs) under different hypotheses (e.g., dispersion, founder-event speciation). Furthermore, BioGeoBears allows for the implementation of a third parameter (J) which permits a “jump speciation” in the daughter lineage, resulting in a possible different area from the direct ancestor. This feature accounts for the biology of *Jassa* species in this study by virtually including anthropogenic dispersal. The analyses were run using the DEC and DEC+J models (Matzke, 2014) and a maximum of five (*J. marmorata*) and four (*J. slatteryi*) areas of possible occurence as this was the maximum number of regions where a single haplotype was found for each species respectively. In order to avoid overinterpretation, only the nodes with at least 70% of bootstrap support and 90% of posterior probabilities were considered. The best-fitting model was selected based on Akaike’s information criterion (AIC).

## Results

The distance between species was 15.2% ± 1.7 and the average distance within species accounted 0.17% ± 0.07 for *J. marmorata* and 0.57% ± 0.17 for *J. slatteryi*.

### Genetic diversity and population structure

The alignment consisted of CO1 sequences from 475 individuals (Table S1). Across the entire data set, 110 sites were identified as polymorphic, 15 were singletons, and 95 were parsimony informative (comprising 36 unique haplotypes). With respect to the overall genetic diversity (all regions combined), *J. marmorata* (19 haplotypes) exhibited lower haplotype diversity (Hd = 0.492) and lower nucleotide diversity (π = 0.002) than *J. slatteryi* (17 haplotypes; Hd = 0.804; π = 0.006) (Table 2).

**Table 2:** Statistical results for *J. marmorata* and *J. slatteryi* for each marine region and sampling location.

	Region	Location	N	H	Hd	π	D	Fs
*Jassa marmorata*	All		390	19	0.492	0.002	**−2.101**	**−11.097**
	Mediterranean Sea	Spain, Mediterranean	18	3	0.523	0.002	0.642	1.974
	NE Pacific	All	109	4	0.305	0.001	−0.621	0.382
		California, USA	78	3	0.378	0.001	0.111	2.001
		Oregon, USA	31	2	0.067	0.000	−1.147	−1.211
	NE Atlantic	All	211	3	0.363	0.001	0.118	0.419
		Spain, Atlantic	8	1	0.000	0.000	0.000	0.000
		North Sea	167	2	0.340	0.001	0.976	1.685
		Norway	16	2	0.533	0.001	1.529	1.362
		Iceland	20	3	0.426	0.001	−0.440	−0.377
	NW Atlantic	All	20	9	0.884	0.006	−0.433	−1.007
		Connecticut, USA	12	7	0.864	0.003	−1.304	−3.413
		New York, USA	2	2	–	–	–	–
		Virginia, USA	4	2	0.500	0.008	−0.829	3.777
		Nova Scotia, Canada	1	1	–	–	–	–
		Maine, USA	1	1	–	–	–	–
	NW Pacific	South Korea	1	1	–	–	–	–
	SE Pacific	All	22	4	0.541	0.003	**−1.504**	1.751
		Chile	14	1	0.000	0.000	0.000	0.000
		Peru	8	3	0.464	0.004	**−1.741**	2.085
	SW Atlantic	Argentina	10	3	0.644	0.001	0.222	−0.046
*Jassa slatteryi*	All		85	17	0.804	0.006	**−0.734**	**−3.251**
	Mediterranean Sea	All	20	6	0.726	0.006	0.324	1.283
		Northern Africa	4	3	0.833	0.006	−0.817	0.961
		Spain, Mediterranean	16	4	0.617	0.004	0.836	1.751
	NE Atlantic	Spain, Atlantic	12	3	0.621	0.006	2.149	4.214
	NE Pacific	California, USA	13	5	0.731	0.005	−0.738	0.865
	NW Atlantic	New York, USA	1	1	–	–	–	–
	NW Pacific	All	17	5	0.680	0.022	−2.469	7.377
		Japan	10	2	0.356	0.001	0.015	0.417
		South Korea	7	4	0.857	0.049	−1.751	5.445
	SE Pacific	All	19	3	0.433	0.002	0.035	1.524
		Chile	18	2	0.366	0.002	0.718	3.088
		Peru	1	1	–	–	–	–
	SW Atlantic	Argentina	2	2	–	–	–	–

**Note:**

Total number of specimens (N), number of haplotypes (H), haplotype diversity (Hd), nucleotide diversity (π), Tajima’s D (D) and Fu’s Fs (Fs). Significant results are highlighted in bold (D confidence level <5%; Fs confidence level <2%). “−” = population with less than three specimens.

Within *J. marmorata*, the haplotype diversity was variable throughout marine regions, ranging from 0.884 in the NW Atlantic region, to 0.305 in the NE Pacific region. Nucleotide diversity was low among the regions, ranging from π = 0.001 in the NE Atlantic to π = 0.006 in the NW Atlantic region.

The haplotype diversity of *J. slatteryi* also exhibited a wide range. The highest regional value of Hd = 0.731 occurred in the NE Pacific (California) while the lowest was detected in the SE Pacific (Hd = 0.433). Nucleotide diversity ranged from π = 0.022 in the NW Pacific to π = 0.002 in the SE Pacific (Table 2).

### Demographic analysis

Tajima’s *D* values were negative and significant for each respective species in total; this indicates an excess of rare nucleotide site variants which suggests a departure from the Wright-Fisher neutral model (Wright, 1931; Table 2). The Fu’s *F*_S_ test, which is based on the distribution of haplotypes, showed a significant negative value for *J. marmorata* in total, indicating an excess of rare haplotypes.

The mismatch distribution analysis was in concordance with *D* and Fs results, for the two species, which supports the spatial and demographic expansion scenarios for both species (Table S2; SSD and Raggedness index).

*Jassa marmorata* did not display departure from the null hypothesis of demographic expansion for any other region except for Peru and the Northern European Seas where no significant spatial expansion was detected (Table S2; SSD and Raggedness index). In contrast, *J. slatteryi* displayed a significant deviation from the null model of demographic expansion for the Mediterranean Sea as well as the North and South East Pacific, whereas there was no significance for a spatial expansion (Table S2; SSD and Raggedness index).

### Geographical structure

The one-way AMOVA revealed significant differences among regions for both species. However, when exploring the variation among populations in the two-way test, the within-region variation was not significant (Table 3). While the main contribution to molecular variance was found within populations, the variation among populations within the same regions was also significant in both species.

**Table 3:** Statistical results of AMOVA tests for *J. marmorata* and *J. slatteryi*.

		Source of variation	df	SS	Variance components		Variation (%)	FCT	FSC	FST
*Jassa marmorata*	One-way	Among regions	5	40.67	0.16	Va	**31.32**			
		Within regions	380	137.01	0.36	Vb	68.68			
		Total	385	177.67	0.52					**0.31323**
	Two-way	Among regions	5	40.67	0.04	Va	8.07	0.08067		
		Among populations	6	27.08	0.19	Vb	**35.81**		**0.38954**	
		Within population	374	109.93	0.29	Vc	**56.12**			**0.43879**
		Total	385	177.67	0.52					
*Jassa slatteryi*	One-way	Among regions	4	34.96	0.47	Va	**28.16**			
		Within regions	75	90.85	1.21	Vb	71.84			
		Total	79	125.81	1.69					**0.28158**
	Two-way	Among regions	4	34.96	−0.12	Va	−7.33	−0.073		
		Among populations	2	12.95	0.74	Vb	**43.91**		**0.409**	
		Within population	73	77.91	1.07	Vc	**63.43**			**0.366**
		Total	79	125.81	1.68					

**Note:**

Significant results (*p* < 0.005) are highlighted in bold.

**Figure 1:**
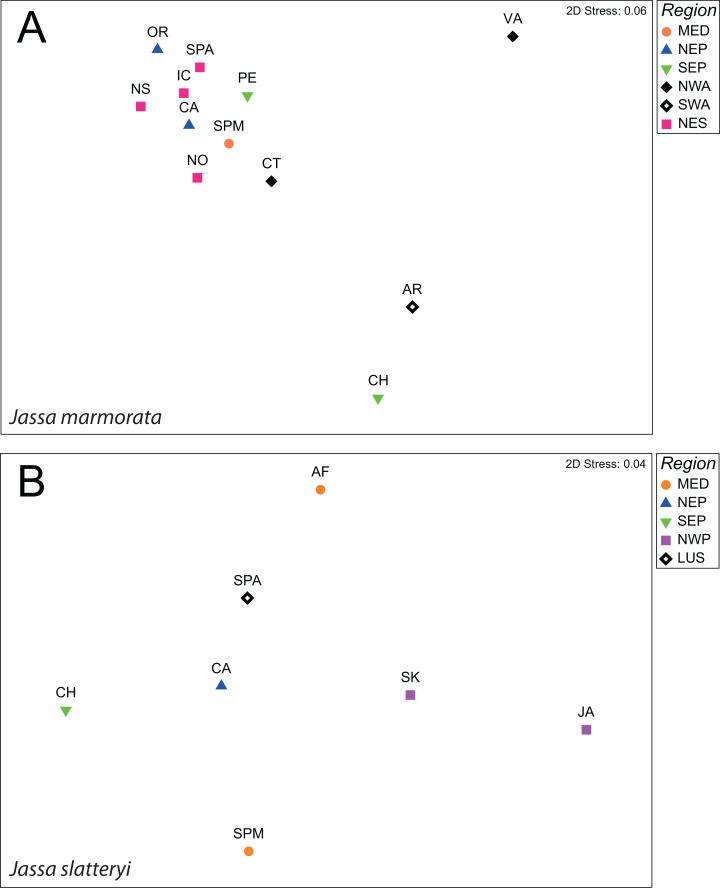
nMDS plots of Fst values between populations *J. marmorata* (A) and *J. slatteryi* (B). Abbreviations: MED = Mediterranean Sea, NEP = North East Pacific, SEP = South East Pacific, NWA = North West Atlantic, SWA = South West Atlantic, NES = Northern European seas, LUS = Iberian Peninsula.

In *J. marmorata*, specimens from the SW Atlantic showed the highest level of differentiation within NE Pacific and Northern European seas (Fst = 0.71 and 0.81 respectively). In the comparison of populations, two main groups were distinguished within *J. marmorata*: a general group with the majority of the populations, and another with Argentina and Chile. The Virginia population showed the highest average differentiation and appeared to be an outgroup (average Fst = 0.776; Fig. 1; Tables S3 and S4).

*Jassa slatteryi* showed an overall low level of differentiation on the regional scale which was only significant for the comparison between Lusitania (Atlantic Spain) and the NE Pacific (California) (Tables S3 and S4). Moreover, there was no obvious pattern with Japan which displayed the highest average Fst (Fst = 0.576; Fig. 1; Table S4).

**Figure 2:**
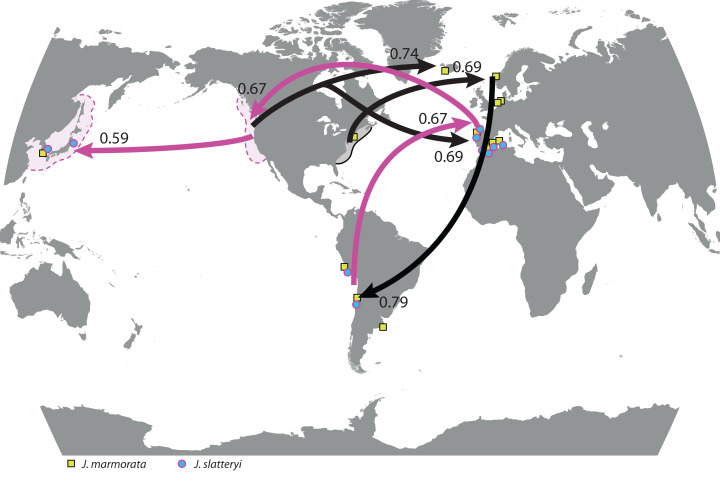
World map with highest significant migration rates. Rates among populations of *J. marmorata* (black) and *J. slatteryi* (purple) from non-adjacent marine regions. Symbols indicate sampling localities for each species made for this study (i.e., excluding sampling localities of previously published sequences). Patterned areas indicate the putative native ranges of *J. mamrorata* (continuous black line) in the North West Atlantic and of *J. slatteryi* (dashed purple lines) in the North Pacific.

For both species, only few migration rates met the assumptions (95% CI > 0; Table S5) and were considered in the migration model among regions. The migration between populations displayed contrasting patterns. In *J. marmorata*, no emigration was observed from Chile, but from almost every other population. In contrast, for Connecticut and Virginia there was only one population donor although multiple receivers were detected (Fig. 2; Tables S5 and S6).

For *J. slatteryi*, neither immigration nor emigration for Mediterranean samples (Spain) and for the NW Pacific populations met the assumptions of the migration model; conversely, more emigrants were found from South Korea to Japan (Fig. 2; Table S5).

**Figure 3:**
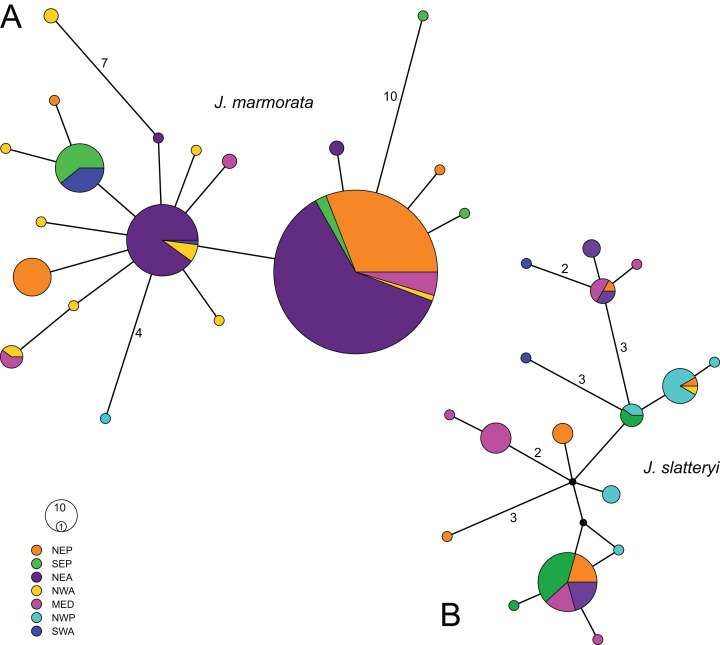
Median joining haplotype networks of *J. marmorata* (A) and *J. slatteryi* (B). Size of the circles are proportional to the number of specimens with relative haplotype. Black dots represent missing haplotypes, more than one mutation is reported with numbers. Abbreviations: NEP = North East Pacific, SEP = South East Pacific, NEA = North East Atlantic, NWA = North West Atlantic, MED = Mediterranean Sea, NWP = North West Pacific, SWA = South West Atlantic.

The haplotype network of *J. marmorata* was star-like with two main haplotypes, one of which occurred in almost every region sampled except the NW Pacific and SW Atlantic (Fig. 3). The second abundant haplotype occurred mostly in the NE Atlantic with additional records from the NW and SW Atlantic. Moreover, only three haplotypes were separated by more than two mutations from either of the two central haplotypes (i.e., SE Pacific: 10, NW Atlantic: 8, NW Pacific: 4), while the highest distance was observed between SE Pacific and NW Atlantic haplotypes (19 steps). On the other hand, the network of *J. slatteryi* was much more dispersed in comparison and did not show any clear pattern of (geographic) segregation but included multiple missing haplotypes and three haplotypes which were present in more than two regions (Fig. 3).

There was no significant correlation between genetic and geographic distances in either *J. marmorata* or *J. slatteryi* (*p* > 0.1).

The phylogeny was poorly resolved, showing high support (posterior probabilities = 1; bootstrap support > 95) for the cluster grouping *J. mamrorata* and *J. slatteryi* as well as each species (Fig. S1).

The DEC+J model significantly increased the likelihood of the DEC model in both *J. marmorata* (InL: DEC+J = −109.76; DEC = −153.97) and *J. slatteryi* (InL: DEC+J = −58.45; DEC = −78.23) (Table S7).

In *J. marmorata*, while the reconstruction of the most recent common ancestor’s (MRCA of all the haplotypes) geographic range was inconclusive, it seemed to exclude the Mediterranean Sea and SW Atlantic but an ancestral divergence of a clade in the NW Atlantic was evident (Fig. S2).

Similar to *J. marmorata*, it was not possible to determine the ancestral geographic range of the MRCA of *J. slatteryi* even though in the Pacific it appeared to be comparatively more diverse. One clade was restricted to the Mediterranean Sea (Fig. S3).

## Discussion

Using the molecular mitochondrial marker CO1, we were able to reconstruct putative native regions (i.e., prior to their “ancient invasion”) of two cosmopolitan amphipods that are common elements of marine biofouling. The examination of the genetic variability between these two species in our analyses indicated high levels of intraspecific divergence within the *J. slatteryi* populations, as well as comparable variation within populations and among populations within regions for *J. marmorata*. A high haplotype number was detected within *J. slatteryi* when compared to *J. marmorata*, and at least two main haplotypes within each species were shared among several geographic populations.

### Genetic population structure and connectivity of *J. marmorata*

The results indicated that the overall percentage of variation present in *J. marmorata* populations was derived almost equally from both variation within populations and variation among populations within marine regions. The highest genetic diversity (accounting for both haplotype and nucleotide diversity values) was found in the NW Atlantic region, and the lowest occurred in the NE Pacific region.

Previous research involving *J. marmorata* samples taken from assumed potential source populations along the Atlantic coast of the United Stated showed varying results: one population from coastal Connecticut revealed no genetic diversity at all in the population, whereas the samples analyzed from coastal South Carolina were shown to have been derived from a separate *Jassa* species-level lineage (Pilgrim & Darling, 2010; J. Beermann, A. Desiderato, 2019–2020, personal observation). Pacific North American coast populations of *J. marmorata* are classified as invasive and are assumed to be transfers from its native range of the Atlantic coast of North America (Chapman, 2007; Conlan, 1990).

In contrast to Pilgrim & Darling (2010), the results of the current study showed high genetic diversity in the presumed native range of *J. marmorata* as well as in populations from the SW Atlantic region. Nevertheless, the presence of a shared haplotype between the SW Atlantic and the SE Pacific region could signal a connection between the two. This is in accordance with the recent first record of *J. marmorata* in Argentina (De Pina, 2005).

Given that introduction events transfer only a subset of native genetic diversity, invasive populations are frequently seen to exhibit lower diversity than the native source populations (Dlugosch & Parker, 2008). The gene pool of introduced populations is expected to be limited due to the stochastic process of introduction mechanisms (Holland, 2000). *J. marmorata*’s low genetic diversity in the NE Pacific is thus consistent with this expected founder effect (Pilgrim & Darling, 2010). Overall haplotype and genetic diversity were lower in *J. marmorata* populations when compared to *J. slatteryi* populations despite a larger sample size. The overall genetic diversity of *J. marmorata* populations was characterized by high haplotype diversity and low nucleotide diversity, indicating only small differences between haplotypes. This is evident from the minimum spanning haplotype network which showed mostly single nucleotide differences between haplotypes in *J. marmorata*.

The obtained data for *J. marmorata* indicated differing levels of geographic structure within the sampled populations; there appeared to be substantial geographic structure within some populations (e.g., Virginia, Chile, Argentina, New York, the North Sea and Oregon) whereas other populations were less structured (Spanish Mediterranean coast and Connecticut). These high levels of structure suggest a more limited gene flow or different selective pressures. Some *J. marmorata* populations were not panmictic throughout a marine region, but displayed high Fst values, indicating an absence of genetic connectivity between them. Populations from Chile-Peru as well as Connecticut-Virginia, on the other hand, could each be assigned to the same marine regions, and were characterized by high heterozygosity values. *J. marmorata* populations from California and Oregon exhibited genetic connectivity with populations in Norway and Iceland, which is unlikely to result from natural dispersal. Furthermore, negative Tajima’s D, Fu’s Fs statistics, as well as the results of the mismatch distributions for *J. marmorata* indicated a population size expansion, perhaps after a bottleneck. It can be assumed that the amount of samples was rather limited considering that the genetic diversity of these populations has probably been shaped largely by stochastic events. A lack of samples from entire marine regions remains an open issue that impedes clear inferences on the connectivity of globally distributed populations.

### Genetic population structure and connectivity of *J. slatteryi*

The magnitude of genetic variation within *J. slatteryi* was highest within populations and lowest among marine regions. In *J. slatteryi*, there was discordance between haplotype and nucleotide diversity. Haplotype diversity was higher in the Mediterranean Sea and the NE Pacific whereas nucleotide diversity was higher in the NW Pacific. The lowest genetic diversity was found in the SE Pacific region.

*Jassa slatteryi* had been previously considered to be either cryptogenic or introduced to the NE Pacific (Boyd, Mulligan & Shaughnessy, 2002). Populations of *J. slatteryi* from the Pacific North American coast had been found to show similar genetic diversity indices, haplotype diversity measures, and intraspecific genetic distances as the presumed native species from that region, *J. staudei* (Chapman, 2007; Pilgrim & Darling, 2010). Both species exhibited diversity measures much higher than *J. marmorata* in the same area. The authors suggested that perhaps the Pacific coast of North America could represent the native range of *J. slatteryi* even though their study was geographically restricted to the continental U.S. coasts.

Our results corroborate the previous assumption that *J. slatteryi* originated from Northern Pacific regions (NE and NW). Surprisingly, there was also high genetic diversity in the populations from the Mediterranean Sea region. The highly divergent haplotypes found in the Mediterranean Sea and the private haplotypes from the SW Atlantic might be artifacts due to small sample sizes and a resulting undersampling in the putative native regions due to the nature of our opportunistic sampling campaign. The overall genetic diversity of *J. slatteryi* populations was different from that of the *J. marmorata* populations as the overall nucleotide diversity was higher, indicating larger differences between haplotypes. This was displayed by the haplotype network where the respective haplotypes were separated by more mutations than in *J. marmorata*.

Populations of *J. slatteryi* also varied in their level of geographic structure, ranging from highly structured populations (Japan) to lower levels of structure (California, Spanish Atlantic coast, Chile and South Korea) and higher gene flow between populations. In contrast to *J. marmorata* populations which showed only limited or no gene flow between populations of the same marine region, *J. slatteryi* populations did not reflect this structure between populations within the same regions. Furthermore, our findings indicate gene flow between geographically remote populations over large distances which is highly unlikely via natural dispersal (e.g., Mediterranean and Spanish Atlantic coasts with South Korea and/or Chile). The mismatch distribution for *J. slatteryi* suggested a geographic expansion but not a demographic expansion.

We identified potential connectivity and dispersal differences between *J. marmorata* and *J. slatteryi* populations in regions where both species co-occurred. *J. slatteryi* populations in Chile, California, South Korea, the Spanish Atlantic and the Mediterranean coasts were particularly closely related to each other (evident by very low Fst values), whereas the *J. marmorata* population from Chile showed more structure with very little gene flow between this and other populations. The Fst values also indicated that, for example, while there is measurable gene flow between *J. marmorata* populations from the neighboring regions of Chile and Argentina, populations of *J. slatteryi* from the same regions are not panmictic. However, both *Jassa* species showed evidence of gene flow between the Mediterranean coast of Spain and California. Both *J. marmorata* and *J. slatteryi* exhibited high haplotype diversity and low nucleotide diversity, indicating rapid demographic expansion from a small effective population size (i.e., contrasting the mismatch results in *J. slatteryi*; Avise, 2000).

The scenario of ongoing dispersal and continuous input of new propagules in different marine regions all over the world is also supported by the inconclusive results of the ancestral range reconstruction of both species, confirming these species’ true identities as “neocosmopolitans”.

### Origin and possible pathways of introduction

Maritime shipping over the past centuries has facilitated dispersal of attached fouling species on the hulls of vessels, and for the last 100± years, transported larvae and other propagules in ballast water around the world. Consequently, this facilitates gene flow between populations, particularly those with non-planktonic larvae (Carlton & Hodder, 1995; Carlton, 1985, 1987; Williams et al., 1988). These unintentional introductions have increased rapidly in recent decades as transit times have diminished, ship-carried trade has become globalized, and ballast-water volumes have risen (Carlton & Geller, 1993; Ruiz et al., 1997; Williams & Grosholz, 2008). Despite the increase in introduction of non-native species by means of maritime traffic, shipping pathways for species dispersal remains poorly understood on a global scale (Mack et al., 2000). Seebens et al. (2013) identified major shipping routes that are high-risk pathways for bioinvasions and also determined that the highest invasion risks were concentrated in a small number of ports. These invasion pathways help explain the geographic distribution of the *Jassa* populations in our study.

The ability of *Jassa* to build tubes on anthropogenic substrata and the absence of planktonic larval stages are excellent prerequisites for human-mediated transport by ships. Accordingly, specimens of both *J. marmorata* and *J. slatteryi* have been reported from hull fouling of ships and boats in different parts of the world (Castro et al., 2020; Martínez-Laiz et al., 2019; Peters, Sink & Robinson, 2019). *J. marmorata* has long been recognized in the literature from worldwide locations (Marchini & Cardeccia, 2017). Although the native origin of *J. marmorata* was uncertain, the worldwide distribution of currently known *Jassa* species indicated that it could be native to the NW Atlantic region where *J. marmorata* occurs as the only species of the genus (Conlan, 1990; Marchini & Cardeccia, 2017). This hypothesis is supported by our findings. Although already Conlan (1990) noted a wide global distribution of *J. slatteryi*, records of this species after Conlan’s original description have increasingly been reported in the past recent years—for example, from Europe (Bonifazi, Mancini & Ventura, 2018; Gouillieux, 2017) or South America (Rumbold et al., 2015). This is probably due to an increasing awareness of this group’s taxonomy, which is corroborated by the standardized utilization of DNA barcoding for species identification. Hence, *J. slatteryi* had been classified as cryptogenic species with uncertain native origin (Marchini & Cardeccia, 2017) but our findings indicate that the species’ likely former native range is the Northern Pacific.

*Jassa* species were most probably transported by ship traffic between NE Atlantic ports and ports in South America (e.g., Argentina, Chile, Peru). The results of this study indicate that these shipping routes played a major role for the establishment of both *J. marmorata* and *J. slatteryi* populations in the SE Pacific and SW Atlantic regions. The models of Seebens et al. (2013) also suggest that although the northern European Seas (NE Atlantic in our study) are recently most strongly connected to tropical and subtropical ecosystems in terms of marine traffic, with the adjacent NW Atlantic waters providing similar enough climatic conditions. Therefore, the North American Atlantic coast still dominates as the major source region of invasions into the NE Atlantic for coastal benthic species. The northern Pacific waters are also source regions for invasions to the NE Atlantic. This could explain the presence of both *J. marmorata* and *J. slatteryi* in the NE Atlantic region. Our data indicate both *Jassa* species occur along the West and East coast of the United States, which is likely due to the connection of both coasts via shipping routes through the Panama Canal. This high invasion risk canal is also passage for shipping routes to and from the NE Atlantic regions (Seebens et al., 2013). The *Jassa* populations from Argentina could have been transported there by way of the Spanish Atlantic and Mediterranean coast, as well as North Africa, and there is a high invasion probability between the NE Pacific region and the SE Pacific region (e.g., Peru and Chile).

## Conclusions

As a result of speculative knowledge of “ancient” invasion events and historically unsettled taxonomy, we cannot determine with certainty through which series of events these *Jassa* species achieved their neocosmopolitan distribution. It is most likely that the species’ distributions are a result of human-mediated introductions which started centuries ago. Our findings indicate that the likely former native range of *J. marmorata* is the NW Atlantic region, and the likely former native range of *J. slatteryi* is the Northern Pacific (NW and NE) region. Due to the relatively fast travel speeds, extensive surface area, and independence of stochastic ocean currents (Haydar, 2012), shipping still appears to be the more successful vector of *Jassa* species dispersal on a global scale as compared to natural dispersal on a local level (e.g., rafting or drifting; Thiel & Haye, 2006). As with these two successful biofoulers, the occurrence of many other coastal benthic species with wide modern geographic distributions is probably the result of human shipping activities over the last centuries.

## Supplemental Information

10.7717/peerj.9613/supp-1 Supplemental Information 1 Phylogenetic time tree of *J. marmorata*, *J. slatteryi* and *J. herdmani* derived with BEAST. Posterior probabilities and bootstrap support are provided when higher than 90% and 70% respectively. Blue bars refer to the 95% highest posterior density intervals for the nodes with at least 90% posterior probabilities

10.7717/peerj.9613/supp-2 Supplemental Information 2 BioGeoBears ancestral range reconstruction under the DEC+J model of *J. marmorata*.. Pie charts at the notes and edges are proportional to the probability of each region before and after the speciation event.

10.7717/peerj.9613/supp-3 Supplemental Information 3 BioGeoBears ancestral range reconstruction under the DEC+J model of *J. slatteryi*. Pie charts at the notes and edges are proportional to the probability of each region before and after the speciation event.

10.7717/peerj.9613/supp-4 Supplemental Information 4 Complete overview of all sequences used for the analyses (collected in this study, and obtained from GenBank and BOLD). Information includes species identity, locality, number of specimens (n), accession numbers, and references.

10.7717/peerj.9613/supp-5 Supplemental Information 5 Sum S (SSD), Raggedness index and relative p-values of the mismatch distributions under the models of demographic expansion and spatial distribution for *J. marmorata* and *J. slatteryi*. Significant *p*-values are highlighted in bold. Regions represented by only one population are annotated in the same line with "/". OR: Oregon; CA: California; NS: North Sea; NO: Norway; IC: Iceland; SPA: Atlantic Spain; CT: Connecticut; VA: Virginia; SPM: Mediterranean Spain; CH: Chile; PE: Peru; JA: Japan; SK: South Korea.

10.7717/peerj.9613/supp-6 Supplemental Information 6 Pairwise Fst indices between regions. Significant Fst values (*p*-value < 0.05) are highlighted in bold. JM: *Jassa marmorata*; JS: *Jassa slatteryi*. NEP: North East Pacific; NES: North european seas; NWA: North West Atlantic; MED: Mediterranean Sea; SEP: South East Pacific; NWP: North West Pacific; LUS: Iberian Peninsula.

10.7717/peerj.9613/supp-7 Supplemental Information 7 Pairwise Fst indices between populations. Significant Fst values (*p*-value < 0.05) are highlighted in bold. JM: *Jassa marmorata*; JS: *Jassa slatteryi*. SPM: Mediterranean Spain; OR: Oregon; CA: California; NS: North Sea; NO: Norway; IC: Iceland; SPA: Atlantic Spain; CT: Connecticut; VA: Virginia; MED: Mediterranean Sea; CH: Chile; PE: Peru; JA: Japan; SK: South Korea; AR: Argentine; AF: Africa.

10.7717/peerj.9613/supp-8 Supplemental Information 8 Migration rates between regions. The source of migration is given in the first row; consistent results are highlighted in bold (always >0). JM: *Jassa marmorata*; JS: *Jassa slatteryi*. NEP: North Eat Pacific; NES: North european seas; NWA: North West Atlantic; MED: Mediterranean Sea; SEP: South East Pacific; NWP: North West Pacific; LUS: Iberian Peninsula.

10.7717/peerj.9613/supp-9 Supplemental Information 9 Migration rates between populations. The source of migration is given in the top row; consistent results are highlighted in bold (always >0). JM: *Jassa marmorata*; JS: *Jassa slatteryi*. OR: Oregon; CA: California; DE: Germany; NS: North Sea; NO: Norway; IC: Iceland; SPA: Atlantic Spain; CT: Connecticut; VA: Virginia; MED: Mediterranean Sea; CH: Chile; PE: Peru; JA: Japan; SK: South Korea.

10.7717/peerj.9613/supp-10 Supplemental Information 10 Summary of likelihood results and the statistics for model comparison with BioGeoBears.

10.7717/peerj.9613/supp-11 Supplemental Information 11 Complete sequence data used for this study. DNA barcode data from all genetically analyzed specimens is also publicly available in the project “JASSA” in BOLD: dx.doi.org/10.5883/DS-JASSA.

## Additional Information and Declarations

### Data Availability

The following information was supplied regarding data availability: DNA barcode data from all genetically analyzed specimens are available in the “JASSA” project at BOLD (DOI 10.5883/DS-JASSA). All sequence data are also available in a Supplemental File. An overview of all used sequences is available in Table S1 including the repository, locality, accession numbers, and references.
