# Supplementary material for: Ancient globetrotters—connectivity and putative native ranges of two cosmopolitan biofouling amphipods
Source: PeerJ. 2020 Jul 28;8:e9613. doi: 10.7717/peerj.9613 (PMC7394068; doi:10.7717/peerj.9613)
Supplement: Supplemental Information 5 — Significant p-values are highlighted in bold. Regions represented by only one population are annotated in the same line with "/". OR: Oregon; CA: California; NS: North Sea; NO: Norway; IC: Iceland; SPA: Atlantic Spain; CT: Connecticut; VA: Virginia; SPM: Mediterranean Spain; CH: Chile; PE: Peru; JA: Japan; SK: South Korea. [file peerj-08-9613-s005.docx]

| *Jassa marmorata* | Demographic expansion | | | | Spatial expansion | | | |
| --- | --- | --- | --- | --- | --- | --- | --- | --- |
|  | SSD | Model (SSD) p-value | Raggedness index | Raggedness p-value | SSD | Model (SSD) p-value | Raggedness index | Raggedness p-value |
| GENERAL | 0.002 | 0.811 | 0.095 | 0.781 | 0.001 | 0.822 | 0.095 | 0.838 |
| Mediterranean Sea/SPM | 0.138 | 0.130 | 0.430 | 0.109 | 0.064 | 0.194 | 0.430 | 0.485 |
| North East Pacific | 0.064 | 0.080 | 0.527 | 0.449 | 0.021 | 0.402 | 0.527 | 0.624 |
| CA | 0.095 | 0.101 | 0.507 | 0.278 | 0.037 | 0.271 | 0.507 | 0.551 |
| OR | 0.000 | 0.185 | 0.756 | 0.871 | 0.000 | 0.223 | 0.756 | 0.809 |
| Northern European Seas | 0.008 | 0.077 | 0.204 | 0.189 | 0.008 | **0.004** | 0.204 | 0.231 |
| NS | 0.003 | 0.133 | 0.219 | 0.202 | 0.003 | **0.006** | 0.219 | 0.239 |
| IC | 0.009 | 0.325 | 0.165 | 0.436 | 0.009 | 0.171 | 0.165 | 0.463 |
| NO | 0.030 | 0.080 | 0.289 | 0.084 | 0.030 | **0.021** | 0.289 | 0.085 |
| North West Atlantic | 0.038 | 0.361 | 0.054 | 0.568 | 0.038 | 0.147 | 0.054 | 0.568 |
| CT | 0.011 | 0.465 | 0.106 | 0.376 | 0.011 | 0.420 | 0.106 | 0.378 |
| South East Pacific | 0.105 | 0.145 | 0.405 | 0.138 | 0.068 | 0.253 | 0.405 | 0.338 |
| PE | 0.283 | **0.001** | 0.228 | 0.979 | 0.041 | 0.479 | 0.228 | 0.782 |
| South West Atlantic/AR | 0.032 | 0.200 | 0.222 | 0.217 | 0.032 | 0.107 | 0.222 | 0.220 |

| *Jassa slatteryi* | Demographic expansion | | | | Spatial expansion | | | |
| --- | --- | --- | --- | --- | --- | --- | --- | --- |
|  | SSD | Model (SSD) p-value | Raggedness index | Raggedness p-value | SSD | Model (SSD) p-value | Raggedness index | Raggedness p-value |
| GENERAL | 0.053 | **0.015** | 0.114 | **0.014** | 0.031 | 0.382 | 0.114 | 0.436 |
| Mediterranean Sea | 0.194 | **0.034** | 0.391 | **0.001** | 0.120 | 0.066 | 0.391 | 0.268 |
| SPM | 0.142 | 0.087 | 0.304 | 0.118 | 0.092 | 0.185 | 0.304 | 0.484 |
| North East Pacific/CA | 0.102 | 0.061 | 0.257 | **0.051** | 0.062 | 0.365 | 0.257 | 0.386 |
| Lusitanian/SPA | 0.171 | 0.078 | 0.249 | 0.132 | 0.080 | 0.215 | 0.249 | 0.723 |
| South East Pacific/CH | 0.172 | **0.048** | 0.670 | 0.313 | 0.086 | 0.131 | 0.670 | 0.545 |
| North West Pacific | 0.034 | 0.400 | 0.119 | 0.577 | 0.030 | 0.436 | 0.119 | 0.680 |
| JA | 0.004 | 0.448 | 0.210 | 0.497 | 0.004 | 0.252 | 0.210 | 0.498 |
| SK | 0.109 | 0.094 | 0.277 | 0.174 | 0.095 | 0.173 | 0.277 | 0.359 |
