# Supplementary material for: Ancient globetrotters—connectivity and putative native ranges of two cosmopolitan biofouling amphipods
Source: PeerJ. 2020 Jul 28;8:e9613. doi: 10.7717/peerj.9613 (PMC7394068; doi:10.7717/peerj.9613)
Supplement: Supplemental Information 6 — Significant Fst values (p-value < 0.05) are highlighted in bold. JM: Jassa marmorata; JS: Jassa slatteryi. NEP: North East Pacific; NES: North european seas; NWA: North West Atlantic; MED: Mediterranean Sea; SEP: South East Pacific; NWP: North West Pacific; LUS: Iberian Peninsula. [file peerj-08-9613-s006.docx]

| *JM* | MED | NEP | NES | NWA | SEP | SWA |
| --- | --- | --- | --- | --- | --- | --- |
| MED |  | 0.006 ± 0.002 | 0.002 ± 0.001 | 0.005 ± 0.003 | 0.000 ± 0.000 | 0.000 ± 0.000 |
| NEP | **0.148** |  | 0.001 ± 0.001 | 0.000 ± 0.000 | 0.000 ± 0.000 | 0.000 ± 0.000 |
| NES | **0.199** | **0.039** |  | 0.000 ± 0.000 | 0.000 ± 0.000 | 0.000 ± 0.000 |
| NWA | **0.130** | **0.405** | **0.524** |  | 0.000 ± 0.000 | 0.000 ± 0.000 |
| SEP | **0.246** | **0.406** | **0.538** | **0.173** |  | 0.011 ± 0.004 |
| SWA | **0.5626** | **0.712** | **0.812** | **0.297** | **0.170** |  |

| *JS* | MED | NEP | LUS | SEP | NWP |
| --- | --- | --- | --- | --- | --- |
| MED |  | 0.020 ± 0.004 | 0.015 ± 0.0031 | 0.001 ± 0.001 | 0.000 ± 0.000 |
| NEP | **0.127** |  | 0.072 ± 0.010 | 0.041 ± 0.007 | 0.000 ± 0.000 |
| LUS | **0.175** | 0.102 |  | 0.014 ± 0.003 | 0.000 ± 0.000 |
| SEP | **0.264** | **0.106** | **0.210** |  | 0.000 ± 0.000 |
| NWP | **0.365** | **0.334** | **0.399** | **0.573** |  |
