# Supplementary material for: Ancient globetrotters—connectivity and putative native ranges of two cosmopolitan biofouling amphipods
Source: PeerJ. 2020 Jul 28;8:e9613. doi: 10.7717/peerj.9613 (PMC7394068; doi:10.7717/peerj.9613)
Supplement: Supplemental Information 7 — Significant Fst values (p-value < 0.05) are highlighted in bold. JM: Jassa marmorata; JS: Jassa slatteryi. SPM: Mediterranean Spain; OR: Oregon; CA: California; NS: North Sea; NO: Norway; IC: Iceland; SPA: Atlantic Spain; CT: Connecticut; VA: Virginia; MED: Mediterranean Sea; CH: Chile; PE: Peru; JA: Japan; SK: South Korea; AR: Argentine; AF: Africa. [file peerj-08-9613-s007.docx]

| *JM* | SPM | CA | OR | SPA | NS | NO | IC | CT | VA | CH | PE | AR |
| --- | --- | --- | --- | --- | --- | --- | --- | --- | --- | --- | --- | --- |
| SPM |  | 0.007 ± 0.003 | 0.003 ± 0.002 | 0.168 ± 0.012 | 0.00293 ± 0.002 | 0.094 ± 0.009 | 0.037 ± 0.006 | 0.081 ± 0.010 | 0.0000 ± 0.000 | 0.000 ± 0.000 | 0.057 ± 0.006 | 0.000 ± 0.000 |
| CA | **0.117** |  | 0.005 ± 0.002 | 0.305 ± 0.015 | 0.000 ± 0.000 | 0.035 ± 0.006 | 0.107 ± 0.009 | 0.002 ± 0.001 | 0.000 ± 0.000 | 0.000 ± 0.000 | 0.030 ± 0.006 | 0.000 ± 0.000 |
| OR | **0.260** | **0.108** |  | 0.999 ± 0.000 | 0.011 ± 0.004 | 0.001 ± 0.001 | 0.006 ± 0.003 | 0.000 ± 0.000 | 0.000 ± 0.000 | 0.000 ± 0.000 | 0.075 ± 0.009 | 0.000 ± 0.000 |
| SPA | 0.117 | 0.045 | -0.062 |  | 0.226 ± 0.011 | 0.026 ± 0.005 | 0.428 ± 0.017 | 0.003 ± 0.002 | 0.003 ± 0.002 | 0.000 ± 0.000 | 0.999 ± 0.000 | 0.000 ± 0.000 |
| NS | **0.203** | **0.062** | **0.121** | 0.070 |  | 0.011 ± 0.003 | 0.250 ± 0.012 | 0.000 ± 0.000 | 0.000 ± 0.000 | 0.000 ± 0.000 | 0.007 ± 0.002 | 0.000 ± 0.000 |
| NO | 0.074 | **0.090** | **0.516** | **0.373** | **0.164** |  | 0.024 ± 0.005 | 0.132 ± 0.013 | 0.000 ± 0.000 | 0.000 ± 0.000 | 0.014 ± 0.003 | 0.000 ± 0.000 |
| IC | **0.105** | 0.037 | **0.099** | 0.010 | 0.012 | **0.176** |  | 0.000 ± 0.000 | 0.000 ± 0.000 | 0.000 ± 0.000 | 0.086 ± 0.010 | 0.000 ± 0.000 |
| CT | 0.084 | **0.251** | **0.558** | **0.358** | **0.445** | 0.054 | **0.305** |  | 0.004 ± 0.002 | 0.000 ± 0.000 | 0.001 ± 0.001 | 0.000 ± 0.000 |
| VA | **0.687** | **0.821** | **0.912** | **0.781** | **0.918** | **0.775** | **0.821** | **0.629** |  | 0.001 ± 0.001 | 0.000 ± 0.000 | 0.000 ± 0.000 |
| CH | **0.653** | **0.672** | **0.977** | **1.000** | **0.836** | **0.812** | **0.863** | **0.561** | **0.854** |  | 0.000 ± 0.000 | 0.003 ± 0.002 |
| PE | 0.114 | **0.153** | 0.175 | 0.000 | **0.291** | **0.204** | 0.091 | **0.233** | **0.599** | **0.703** |  | 0.001 ± 0.001 |
| AR | **0.563** | **0.647** | **0.899** | **0.826** | **0.822** | **0.671** | **0.760** | **0.438** | **0.740** | **0.430** | **0.558** |  |

| *JS* | SP | AF | CA | SPA | CH | JA | SK |
| --- | --- | --- | --- | --- | --- | --- | --- |
| SP |  | 0.001 ± 0.001 | 0.005 ± 0.002 | 0.000 ± 0.000 | 0.000 ± 0.000 | 0.000 ± 0.000 | 0.003 ± 0.002 |
| AF | **0.528** |  | 0.021 ± 0.004 | 0.198 ± 0.015 | 0.005 ± 0.002 | 0.000 ± 0.000 | 0.015 ± 0.003 |
| CA | **0.237** | **0.331** |  | 0.081 ± 0.009 | 0.047 ± 0.007 | 0.000 ± 0.000 | 0.041 ± 0.006 |
| SPA | **0.336** | 0.052 | 0.102 |  | 0.018 ± 0.004 | 0.000 ± 0.000 | 0.018 ± 0.005 |
| CH | **0.394** | **0.618** | **0.106** | **0.210** |  | 0.000 ± 0.000 | 0.001 ± 0.001 |
| JA | **0.627** | **0.676** | **0.475** | **0.490** | **0.740** |  | 0.007 ± 0.003 |
| SK | **0.354** | **0.376** | **0.145** | **0.250** | **0.447** | **0.310** |  |
