# Supplementary material for: Ancient globetrotters—connectivity and putative native ranges of two cosmopolitan biofouling amphipods
Source: PeerJ. 2020 Jul 28;8:e9613. doi: 10.7717/peerj.9613 (PMC7394068; doi:10.7717/peerj.9613)
Supplement: Supplemental Information 8 — The source of migration is given in the first row; consistent results are highlighted in bold (always >0). JM: Jassa marmorata; JS: Jassa slatteryi. NEP: North Eat Pacific; NES: North european seas; NWA: North West Atlantic; MED: Mediterranean Sea; SEP: South East Pacific; NWP: North West Pacific; LUS: Iberian Peninsula. [file peerj-08-9613-s008.docx]

| *JM* | NEP | NES | NWA | MED | SEP |
| --- | --- | --- | --- | --- | --- |
| NEP |  | 1402.5 | 1372.5 | 1192.5 | 1252.5 |
| NES | 1462.5 |  | **1657.5** | 982.5 | 637.5 |
| NWA | 382.5 | 487.5 |  | 997.5 | 622.5 |
| MED | **1807.5** | **1582.5** | **1477.5** |  | 1057.5 |
| SEP | 1417.5 | 1252.5 | 1342.5 | 1042.5 |  |

| *JS* | NEP | SEP | NWP | MED | LUS |
| --- | --- | --- | --- | --- | --- |
| NEP |  | **1597.5** | 1057.5 | 1162.5 | 1387.5 |
| SEP | 1372.5 |  | 937.5 | 1222.5 | 1327.5 |
| NWP | 1477.5 | 667.5 |  | 1057.5 | 787.5 |
| MED | 1162.5 | 1192.5 | 562.5 |  | 1417.5 |
| LUS | 1327.5 | **1672.5** | 892.5 | 1342.5 |  |
