# Supplementary material for: Ancient globetrotters—connectivity and putative native ranges of two cosmopolitan biofouling amphipods
Source: PeerJ. 2020 Jul 28;8:e9613. doi: 10.7717/peerj.9613 (PMC7394068; doi:10.7717/peerj.9613)
Supplement: Supplemental Information 9 — The source of migration is given in the top row; consistent results are highlighted in bold (always >0). JM: Jassa marmorata; JS: Jassa slatteryi. OR: Oregon; CA: California; DE: Germany; NS: North Sea; NO: Norway; IC: Iceland; SPA: Atlantic Spain; CT: Connecticut; VA: Virginia; MED: Mediterranean Sea; CH: Chile; PE: Peru; JA: Japan; SK: South Korea. [file peerj-08-9613-s009.docx]

| *JM* | OR | CA | DE | NS | NO | IC | SPA | CT | VA | MED | CH | **PE** |
| --- | --- | --- | --- | --- | --- | --- | --- | --- | --- | --- | --- | --- |
| OR |  | 1432.5 | 1162.5 | **1612.5** | 1402.5 | 1447.5 | 1372.5 | 1312.5 | 1357.5 | 1432.5 | 1372.5 | **1552.5** |
| CA | **1552.5** |  | 1132.5 | **1537.5** | 1447.5 | 1447.5 | **1567.5** | **1582.5** | **1522.5** | 1357.5 | 1327.5 | **1582.5** |
| DE | 1147.5 | 1147.5 |  | 1087.5 | **1522.5** | 1462.5 | 1507.5 | **1582.5** | **1552.5** | 1312.5 | 1042.5 | **1537.5** |
| NS | **1522.5** | 1507.5 | 1237.5 |  | 1402.5 | 1387.5 | 1402.5 | 1417.5 | **1552.5** | 1447.5 | 1327.5 | 1447.5 |
| NO | 1282.5 | 1162.5 | **1732.5** | 1192.5 |  | **1567.5** | 1402.5 | **1642.5** | **1537.5** | 1162.5 | 1012.5 | **1507.5** |
| IC | **1657.5** | **1507.5** | 1372.5 | **1627.5** | **1597.5** |  | 1432.5 | 1312.5 | 1432.5 | 1282.5 | 1177.5 | **1522.5** |
| SPA | **1642.5** | **1552.5** | 1222.5 | **1672.5** | 1477.5 | **1582.5** |  | 1492.5 | **1582.5** | 1372.5 | 1447.5 | 1447.5 |
| CT | 1057.5 | 1207.5 | 1402.5 | 1027.5 | **1582.5** | 1387.5 | 1477.5 |  | 1432.5 | 1477.5 | 952.5 | 1327.5 |
| VA | 1132.5 | 1192.5 | 1027.5 | 1042.5 | 1387.5 | 1357.5 | 1387.5 | 1357.5 |  | **1612.5** | 997.5 | 1432.5 |
| MED | **1507.5** | 1387.5 | 1147.5 | **1522.5** | 1492.5 | 1402.5 | 1447.5 | 1402.5 | 1417.5 |  | 1372.5 | **1507.5** |
| CH | 1432.5 | **1507.5** | **1462.5** | 1402.5 | **1672.5** | **1522.5** | **1582.5** | **1552.5** | **1597.5** | **1597.5** |  | **1537.5** |
| PE | 1417.5 | 1462.5 | 1132.5 | 1492.5 | 1417.5 | **1492.5** | 1462.5 | **1612.5** | **1687.5** | 1357.5 | 1417.5 |  |

| *JS* | CA | CH | JA | SK | MED | SPA |
| --- | --- | --- | --- | --- | --- | --- |
| CA |  | **1477.5** | 1012.5 | 1402.5 | 1057.5 | **1567.5** |
| CH | **1537.5** |  | 1117.5 | **1507.5** | 1327.5 | 1417.5 |
| JA | **1552.5** | 1102.5 |  | **1627.5** | 1297.5 | 1282.5 |
| SK | 1312.5 | 742.5 | **1447.5** |  | 937.5 | 937.5 |
| MED | 1342.5 | 1237.5 | 742.5 | 1327.5 |  | 1117.5 |
| SPA | 1432.5 | **1567.5** | 952.5 | 1357.5 | 1207.5 |  |
