# Supplementary material for: Ancient globetrotters—connectivity and putative native ranges of two cosmopolitan biofouling amphipods
Source: PeerJ. 2020 Jul 28;8:e9613. doi: 10.7717/peerj.9613 (PMC7394068; doi:10.7717/peerj.9613)
Supplement: Supplemental Information 10 [file peerj-08-9613-s010.docx]

| Species | alt.model | null.model | LnL.alt | LnL.null | DF.alt | DF.null | DF | D.statistic | p-value | test | tail | AIC.null | AIC.alt |
| --- | --- | --- | --- | --- | --- | --- | --- | --- | --- | --- | --- | --- | --- |
| *Jassa marmorata* | DEC+J | DEC | -109.8 | -154.0 | 3 | 2 | 1 | 88.42 | **5.30E-21** | chi-squared | one-tailed | 225.5 | 311.9 |
| *Jassa slatteryi* | DEC+J | DEC | -58.45 | -78.23 | 3 | 2 | 1 | 39.57 | **3.20E-10** | chi-squared | one-tailed | 122.9 | 160.5 |
